# Habitual Physical Activity in Mitochondrial Disease

**DOI:** 10.1371/journal.pone.0022294

**Published:** 2011-07-22

**Authors:** Shehnaz Apabhai, Grainne S. Gorman, Laura Sutton, Joanna L. Elson, Thomas Plötz, Douglass M. Turnbull, Michael I. Trenell

**Affiliations:** 1 Mitochondrial Research Group, Newcastle University, Newcastle upon Tyne, United Kingdom,; 2 NIHR Biomedical Research Centre for Ageing and Age-related Disease, Newcastle University, Newcastle upon Tyne, United Kingdom; 3 Newcastle Centre for Brain Ageing and Vitality, Newcastle University, Newcastle upon Tyne, United Kingdom; 4 School of Computing, Newcastle University, Newcastle upon Tyne, United Kingdom; Charité Universitätsmedizin Berlin, NeuroCure Clinical Research Center, Germany

## Abstract

**Purpose:**

Mitochondrial disease is the most common neuromuscular disease and has a profound impact upon daily life, disease and longevity. Exercise therapy has been shown to improve mitochondrial function in patients with mitochondrial disease. However, no information exists about the level of habitual physical activity of people with mitochondrial disease and its relationship with clinical phenotype.

**Methods:**

Habitual physical activity, genotype and clinical presentations were assessed in 100 patients with mitochondrial disease. Comparisons were made with a control group individually matched by age, gender and BMI.

**Results:**

Patients with mitochondrial disease had significantly lower levels of physical activity in comparison to matched people without mitochondrial disease (steps/day; 6883±3944 vs. 9924±4088, p = 0.001). 78% of the mitochondrial disease cohort did not achieve 10,000 steps per day and 48%were classified as overweight or obese. Mitochondrial disease was associated with less breaks in sedentary activity (Sedentary to Active Transitions, % per day; 13±0.03 vs. 14±0.03, p = 0.001) and an increase in sedentary bout duration (bout lengths / fraction of total sedentary time; 0.206±0.044 vs. 0.187±0.026, p = 0.001). After adjusting for covariates, higher physical activity was moderately associated with lower clinical disease burden (steps / day; r_s_ = −0.49; 95% CI −0.33, −0.63, P<0.01). There were no systematic differences in physical activity between different genotypes mitochondrial disease.

**Conclusions:**

These results demonstrate for the first time that low levels of physical activity are prominent in mitochondrial disease. Combined with a high prevalence of obesity, physical activity may constitute a significant and potentially modifiable risk factor in mitochondrial disease.

## Introduction

Mitochondria are ubiquitous intracellular organelles found in all nucleated cells and are responsible for energy production and cellular respiration. They are involved in various different cellular processes including calcium signalling [1], cellular metabolism [2], and cytochrome *c* mediated apoptosis [3]. Mitochondrial enzymes are integral in intermediary metabolism [4]; but, it is usually defects in the respiratory chain, the common final pathway of energy metabolism that result in what are termed mitochondrial cytopathies [5]. Mitochondrial disorders typically affect organs that are heavily dependent on oxidative metabolism such as the brain, eye and skeletal muscle and consequently are commonly characterised by multi-system involvement and variable phenotypic expression, disease severity and rate of progression [6], [7], [8]. Mitochondrial disease is the most common neuromuscular disease [9] and there are currently no validated therapies.

Exercise intolerance is a characteristic hallmark of patients with mitochondrial disease, with muscle weakness and fatigue reported after low levels of exertion [10]. It is not known whether the reduced exercise tolerance associated with mitochondrial disease is associated with reduced daily physical activity. As an important mediator of mortality, life expectancy, physical function and the onset of disability in normal aging [11], [12], [13], physical activity may represent an environmental influence that acts independently of the genetic predisposition.

Several studies have now shown that the reversal of a sedentary lifestyle in mitochondrial disease with exercise therapy confers benefits to mitochondrial function and have confirmed safety [14], [15], [16], [17]. Before critically evaluating exercise as a potential therapy in mitochondrial disease it is important to understand the additional risks that patients with mitochondrial disease may be exposed to as a result of low levels of habitual physical activity. Consequently, the aims of this study were to characterise habitual physical activity in patients with known mitochondrial disease and evaluate its relationship with genotype and phenotype.

## Methods

### Subjects

One hundred patients with a diagnosis of mitochondrial disease were enrolled in the study. Forty-seven participants had a diagnosed point mutation (29 patients with m.3243A>G MELAS mutation; 8 patients with m.8344A>G MERRF mutation: 9 patients with other mitochondrial tRNA (mt-tRNA) mutations). Twenty-eight participants were diagnosed with multiple mtDNA deletions (15 patients unspecified nuclear genetic defect, three patients OPA1 (optic atrophy type 1), five patients POLG1 (polymerase gamma) and five patients PEO1 (Twinkle)). Twenty-one participants were diagnosed with heteroplasmic single-large scale mtDNA deletion. The remaining four patients had uncharacterised genetic defects, but presence of ragged red fibres and Cox negative staining from their biopsy (1–3%). From the patient group 1 participant was wheelchair bound and 1 used a wheel chair for mobilization outside the home. None of the patients were taking part in any exercise based interventions during the duration of the trial. Two of the patients had clinically identified mild cardiomyopathy but without exercise limitation, none had undergone formal evaluation of liver function. All patients had cognitive function sufficient for provision of informed consent (Mini-Mental State Examination >26). The control group consisted of 100 participants, individually matched by age, gender and body mass index (BMI). Participants were over 18 years of age and gave written informed consent. All participants provided informed consent and the study was approved by the Newcastle regional medical research ethics committee.

### Physical Activity

Habitual physical activity was measured objectively using a validated multi-sensor array [18] (SenseWear Pro_3_, Bodymedia Inc, PA, USA) worn over 3 days. Physical activity data are presented as average METs / day, where 1 MET  =  resting metabolic rate, or in steps / day. Patterns of sedentary behaviour were assessed by power law analyses of the lengths of sedentary bouts fitted from raw sedentary data, as described in more detail previously [19]. Briefly; the density *p*(*x*) of sedentary bouts in a time bin width *d*(*x*) was plotted against the bout length *x* on a logarithmic scale to derive power distribution (equation 1) from the shape of the histogram with respect to there length_ α_:
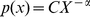
(1)


The type of sedentary distribution characterised by the exponent _α_ (equation 2), can quantify different sedentary behaviour strategies, with a lower _α_ indicating that subjects accumulate sedentary time with a larger proportion of long sedentary bouts:
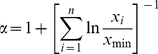
(2)


From these power distributions, Lorentz curves were calculated where the fraction *Wx* of the total sedentary time that is accumulated in bouts longer than any sedentary period of length *x*:
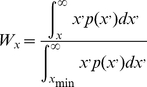
(3)


The curves are then plotted as *Wx* / *p*(*x*) pairs for each patient and control. Activity patterns were also assessed by assessing transitions from being inactive to active and normalized by the length of the recording, termed “Sedentary to Active Transitions”. These data are presented as a percentage of the activity data per day. Subjective reports of habitual physical activity were assessed using the International Physical Activity Questionnaire (IPAQ) long form [20].

### Clinical Presentations

Disease severity was assessed using the Newcastle Mitochondrial Disability Adult Scale (NMDAS) [21]. The NMDAS is a validated clinical rating scale consists of four domains: current function; system specific involvement; current clinical assessment; and quality of life. Each item within these domains is allocated one question with a six-point response scale scored between 0 (unaffected) and 5(severely affected). A low NMDAS score is advantageous and a high NMDAS score is disadvantageous for the patient. This tool semi-quantifies disease severity and longitudinally, can evaluate the long-term natural progression of mitochondrial myopathies.

### Statistical Analysis

Assessment of normality was performed using a Shapiro Wilks Test and skewed data were transformed using natural logarithms or analysed using rank tests. Differences between patient and control group values were calculated and analysed using paired t-tests. The relationships between physical activity and clinical presentations in the patient group were determined using hierarchical regression, controlling for age, gender and BMI. Relationships between physical activity and BMI in patient and control groups were also determined using regression models, adjusted for age, gender and, where appropriate, disease severity. Variables included in the NMDAS were assessed using correlation and regression techniques to determine their relative contribution to the relationship between physical activity and disease severity. Assumption and diagnostic checks were performed on all data. All statistical analyses were performed using SPSS for Windows, version 16 (SPSS Inc., Illinois, USA). All values are expressed as mean ± SD unless otherwise stated.

## Results

### Baseline characteristics

General group descriptions stratified by genotype can be found in Table 1. Habitual physical activity was low, with 78% of the patient cohort taking less than 10,000 steps per day. Twenty-eight per cent of the mitochondrial disease group were overweight (BMI 25–29.9 kg/m^2^) and 20% were obese (BMI ≥30 kg/m^2^); only 4% were underweight (BMI<18.5 kg/m^2^). Patient characteristics (gender, age, BMI, number of steps walked per day, average METS and NMDAS score) were similar across genotype categories.

**Table 1:** Clinical, physical, and descriptors of habitual physical activity for all study participants and for subgroups by genetic mutation.

*Genotype*	N	Age	BMI	Clinical Rating (NMDAS)	Average Daily METS	Number of Steps Walked Per Day	Subjective recall of Physical activity (met.min.wk)
Point Mutations	47	47±12	25±6	21±14	1.4±0.3	6967±3760	2344±5253
Multiple Deletions	28	56±11	27±5	28±16	1.4±0.3	7162±4187	3414±1466
Single deletion	21	49±13	25±5	23±15	1.3±0.2	6360±3745	2125±2347
Complex deficiency	4	51±14	23±4	20±24	1.4±0.4	6715±7055	5494±5385
**All patients**	100	50±12	26±7	23±15	1.4±0.3*	6883±3944*	3356±5531
**Control group**	100	50±13	25±4	-	1.5±0.2	9924±4088	-

Data are expressed as Mean ± SD. *  =  significantly different from control group.

### Age, gender, disease burden and physical activity

**Figure 1:**
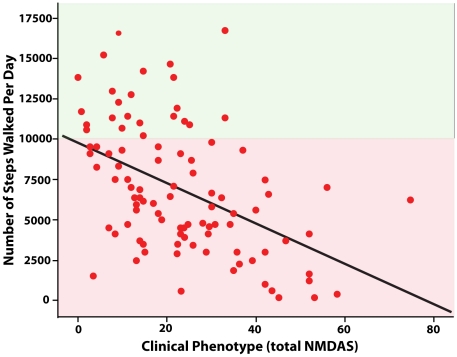
Scatter plot of the relationship between disease burden (total NMDAS score) and habitual physical activity (steps walked per day) (unadjusted regression line, R-Sq = 0.22, *p*<0.001). The red zone represents under and green zone over 10,000steps per day; the advised level of physical activity by the World Health Organisation [22] for health.

Mitochondrial disease patients performed significantly less physical activity than the control group (Table 1), with mean differences between groups of 3041 steps per day (95% CI 1966, 4117) and 0.09 average METs per day (95% CI 0.02, 0.16). A higher disease burden assessed by the NMDAS was associated with a lower level of physical activity (Figure 1, steps / day; r_s_ = −0.49; 95% CI −0.33, −0.63, P<0.01, avMETs / day; r_s_ = −0.30; 95% CI −0.11, −0.47, p<0.01). After partitioning the variance accounted for by age, gender and BMI, the variance in physical activity (METs and steps, respectively) explained by disease severity was 4 – 15%.

The NMDAS subscales showing significant correlations with physical activity variables were entered into exploratory regression models; the only variable to be retained in all models following stepwise iterations was the exercise subscale, with R^2^ values of 25% when steps per day was the response variable and 19% for METs. Physical activity as measured by METs was negatively associated with BMI in both patient and control groups, with a stronger relationship evident in the control group. In the patient group, METs was a significant predictor (β = −0.32, p = 0.002) of BMI and accounted for 10.3% of the variance after partialling out the effects of age, gender and disease severity. After adjusting for age and gender, METs was a significant predictor (β = −0.67, p<0.001) of, and accounted for 34.8% of the variance in, BMI in the control group. Physical activity as measured by steps per day was not a significant predictor of BMI in either patient or control groups.

Low levels of physical activity were common in the patient group, with half of the group having an average daily energy expenditure of less than 1.4 times their resting metabolic rate. Objective measures of physical activity were seen to be moderately associated with subjective reports of physical activity at work, leisure time, active transport (steps variable only) and with the amount of time (min) spent sitting per day (0.25<r_s_<0.35; lowest 95% confidence limit 0.05, highest 0.51). Objective measures of physical activity were not associated with subjective reports during domestic & gardening activities.

### Sedentary Activity

**Figure 2:**
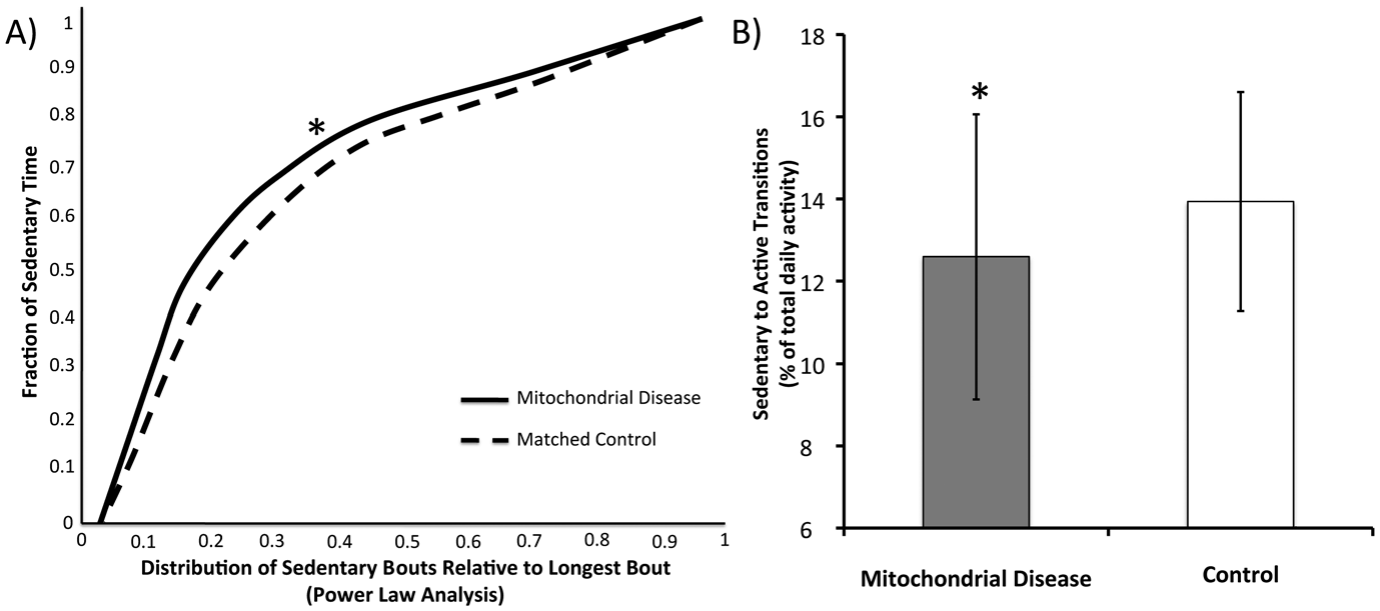
Power law analyses of the lengths of sedentary bouts of patients with mitochondrial disease [line] and controls [dashed] (Panel A). Sedentary to Active Transitions, indicating transition from inactive to active periods, in patients with mitochondrial disease [grey] and controls [open] (Panel B). Data are Mean ± SD. *  =  significantly different from control p = 0.007.

Power law analyses of the lengths of sedentary bouts demonstrate that patients with mitochondrial disease have a longer duration of sedentary bout (Figure 2A, Mean difference 0.02; 95% CI 0.01, 0.03, t_74_ = 3.32, p = 0.001). The number of transitions from being sedentary to activity were significantly lower in patients with mitochondrial disease compared with controls (Figure 2B, Mean difference −0.01; 95% CI −0.02, −0.00, t_75_ = −2.78, p = 0.007).

## Discussion

This is the first study to report daily physical activity levels of people with mitochondrial disease. The major findings of this study are: 1) a sedentary lifestyle is prominent in people with mitochondrial disease, 2) across all genotypes there is a significant inverse relationship between clinical rating and measures of physical activity, with patients with greater phenotypic disease burden undertaking less daily physical activity; and 3) 48% of the patient group were either overweight or obese; only 4% were underweight.

The first key observation was the low level of physical activity in the patient cohort; with 78% of the patients not achieving internationally advised levels of physical activity (10,000 steps per day [22]). The failure to achieve advised levels of physical activity is acknowledged to have an adverse influence upon healthy living, compounding metabolic diseases and reducing life expectancy [12], [23], [24]. Recent studies have suggested that sedentary behaviour, in contrast to active energy expenditure, is possibly a more important predictor of metabolic risk though its significant impact on metabolic regulation [24], [25]. In this light, analysis of the raw physical activity data reveals that the average duration of sedentary periods is longer in patients with mitochondrial disease than controls. Breaks in sedentary time are also fewer than controls. Combined, these data suggest that patients with mitochondrial disease are at an increased risk of metabolic disease due to their low levels of physical activity and prominent sedentary behaviour, independent of their genetic predisposition.

Clinical rating was inversely related to habitual physical activity, with those undertaking less physical activity having greater physical impairment. The relationship between clinical rating and habitual physical activity is intuitive and follows similar relationships demonstrated in other metabolic disorders [24]. The large variation in physical activity data for people with similar disease burden also provides an insight into the ability of people with mitochondrial disease to maintain a physically active lifestyle. There are individuals with low clinical ratings (<4) who have no clinical reason to have low levels of physical activity, yet have very low levels of habitual physical activity (<2000 steps per day / <1.3 METS/day [Figure 1]). Conversely, there are those with high clinical ratings who do have significant physical impairment, yet achieve high levels of physical activity. As such, although clinical phenotype is broadly related to physical activity it is clear that clinical phenotype alone *does not* dictate the level of physical activity. This observation is of particular interest as several studies have now shown that the reversal of a sedentary lifestyle with exercise therapy confers benefits to mitochondrial function and have confirmed safety [14], [15], [16], [17].

Additional information can be gleaned by analysis of the sub-scales of the NMDAS and the subjective reports of physical activity. Clinical sub-scales associated with physical function, such as cerebellar dysfunction and exercise intolerance were associated with low levels of physical activity. Again, there is significant breadth in the clinical presentations within these scales – with some phenotypically healthy individuals demonstrating low levels of physical activity and vice versa. There was no association between habitual physical activity and the subscales of myopathy or neuropathy as may have been expected. This may in part be due to the mild nature of both parameters in this cohort. These observations suggest that disease burden is a strong predictor of habitual physical activity but is not the only determinant.

Subjective reports of physical activity provide information about where in everyday life people are physically active and where physical activity is reduced. These data reveal that work and leisure time physical activities were a key determinant of overall physical activity. Not surprisingly, low levels of daily physical activity are associated with high levels of sitting. These observations suggest that some of the key clinical presentations are associated with physical activity and that physical activity, in turn, is reduced in parts of the day where the patients'ave choice of movement, that is, work, leisure an d sitting. The high level of sitting time associated with mitochondrial disease is particularly of concern as high levels of sitting time are associated with a 2.5 times greater risk of heart disease and the development of metabolic syndrome [12], [24]. The next challenge is to better understand how these areas of life can be targeted to increase physical activity, with a view to improving clinical features and reducing metabolic disease risk.

Almost half of the patients were overweight and one in five classified as obese [22]. This is contrary to historical descriptions of svelte body habitus of mitochondrial disease, particularly in relation to the 3243 A>G genotype [26]. This would suggest that people with mitochondrial disease are at added risk of high morbidity and mortality as posed by obesity and low physical activity in addition to the inherent predispositions that accompany mitochondrial disease.

These results show that the majority of people with mitochondrial disease lead an essentially sedentary lifestyle and that low levels of physical activity are related to clinical correlates of function and obesity. Combined, these observations suggest that low levels of physical activity, alongside a high prevalence of obesity, constitute significant disease risk factors in addition to the inherent predispositions that accompany mitochondrial disease and may represent a significant therapeutic target.
